# Co-creation of human papillomavirus self-sampling delivery strategies for cervical cancer screening in rural Zimbabwe: nominal group technique

**DOI:** 10.3389/fpubh.2023.1275311

**Published:** 2023-11-16

**Authors:** Mathias Dzobo, Tafadzwa Dzinamarira, Grant Murewanhema, Tatenda Chishapira, Racheal S. Dube Mandishora, Megan Fitzpatrick, Tivani Mashamba-Thompson

**Affiliations:** ^1^School of Health Systems and Public Health, Faculty of Health Sciences, University of Pretoria, Pretoria, South Africa; ^2^Centre for International Programmes Zimbabwe Trust, Harare, Zimbabwe; ^3^Unit of Obstetrics and Gynaecology, Faculty of Medicine and Health Sciences, University of Zimbabwe, Harare, Zimbabwe; ^4^Medical Microbiology Unit, Department of Laboratory Diagnostics and Investigative Sciences, University of Zimbabwe Faculty of Medicine and Health Sciences, Harare, Zimbabwe; ^5^Moffitt Cancer Center, Center for Immunization and Infection Research in Cancer (CIIRC), Tampa, FL, United States; ^6^Department of Pathology and Laboratory Medicine, University of Wisconsin School of Medicine and Public Health, Madison, WI, United States

**Keywords:** cervical cancer, HPV self-sampling, co-creation, delivery strategies, nominal group technique, Zimbabwe

## Abstract

**Background:**

Human papillomavirus (HPV) self-sampling is recommended for cervical cancer screening, particularly among women who do not participate in or have access to current screening methods offered in Zimbabwe. Key stakeholder involvement is critical in co-creating acceptable delivery strategies for implementing HPV self-sampling to ensure demand and facilitate uptake by the target population. The main objective of this study was to engage key stakeholders in co-creating acceptable HPV self-sampling delivery strategies for cervical cancer screening in rural Zimbabwe.

**Methods:**

We invited key stakeholders and employed a nominal group technique (NGT) for data collection. We employed the NGT to (1) identify barriers to access and utilisation of available cervical cancer screening services and (2) co-create delivery strategies for HPV self-sampling. The workshop included 8 participants (women *n* = 4, health workers *n* = 2 and policymakers *n* = 2). Quantitative data was gathered by ranking ideas and qualitative data were collected from participant group discussions and analysed thematically. The results of the ranking exercise were fed back to the participants for comments.

**Results:**

The most significant barriers to accessing and utilising current cervical cancer screening services by women were: Inadequate information and education on cervical cancer, lack of resources and funding for cervical cancer programmes, long distances to nearest health facilities, and low perceived personal risk of cervical cancer. Key stakeholders recommended enhanced education and awareness, results notification, linkage to care, community-based self-sampling, and the choice of sampling devices as potential HPV self-sampling delivery strategies.

**Conclusion:**

Our study demonstrated the utility of the NGT for reaching a consensus. Using the NGT, we established priority delivery strategies for HPV self-sampling cervical cancer screening. Adequate education and awareness, early results notification, choice of sampling device and community-based self-sampling were crucial to HPV self-sampling screening in rural Zimbabwe. The proposed delivery strategies can guide the development of guidelines for designing and implementing an HPV self-sampling intervention. We recommend a study to determine women's most preferred HPV self-sampling delivery strategies before implementing the intervention.

## Background

Despite being preventable through HPV vaccination, cervical cancer screening, and treatment of cervical precancer, cancer of the cervix is a significant public health challenge in the world. It is the fourth leading cause of cancer deaths among women globally. In 2020 an estimated 604, 000 women were diagnosed with cervical cancer and 342, 000 women died from the disease (1). Compared to high-income countries, low-middle-income countries (LMICs) are disproportionately affected. According to the World Health Organisation (WHO), 19 of the top 20 countries with the highest cervical cancer incidence are in Africa (2). Zimbabwe has one of the highest global mortality rates for cervical cancer with an estimated age-standardised mortality rate of (43.0/100000) which is remarkably higher than the global average of 13.3/100,000 (3). An estimated 3043 women were diagnosed with cervical cancer, and 1976 lost their lives to it in 2020 alone (3). The burden of cervical cancer is compounded by the high prevalence of HIV in the country. According to the last national survey conducted in 2020, the prevalence of HIV among women aged 15 years and older was 15.3% (4). HIV infection is known to increase the risk of developing cervical cancer by up to six-fold (5), making HIV and cervical cancer important public health problems for Zimbabwe.

The marked difference in incidence and mortality between developing countries such as Zimbabwe and high-income countries is largely due to the lack of organised cervical screening using cytology. Similar to other LMICs, Zimbabwe's cervical cancer screening programme using cytology failed to reduce the incidence of cervical cancer due to lack of funding, infrastructure, trained personnel and financial resources (6, 7). Currently, visual inspection with acetic acid and cervicography (VIAC) forms the basis for the majority of cervical cancer screening in Zimbabwe and it is available at 14% of all government health facilities (8). Although available at some of the health facilities, the country's screening coverage remains low with the majority of women never screened and presenting with advanced disease (9, 10). An estimated 20% of all eligible women are ever screened in their lifetime for cervical cancer in Zimbabwe (8). In addition to limited access and unavailability of screening services, other barriers are responsible for preventing women from accessing and utilising available screening services.

Several factors at the individual, interpersonal, community and health system level have been established as barriers to access and utilisation of services. Nyamambi and colleagues identified barriers at the intrapersonal, sociocultural, and health system levels and the lack of education was credited as the most significant individual barrier to the uptake of cervical cancer screening by women (11). Another study conducted in Zimbabwe by Mapanga et al. further reinforced the role of individual factors as significant barriers to the uptake of screening services with the lack of knowledge and awareness of cervical cancer being the most common barrier (12). The same study found that economically disadvantaged women were less likely to seek screening services, which disproportionately affects rural women in Zimbabwe (12).

Besides the primary prevention of vaccinating girls who have never had sex, the WHO recommends the secondary prevention of cervical cancer by HPV testing in LMICs where there are enough resources. The WHO aims to achieve a screening coverage of 70% using HPV testing by 2030 by screening women twice at age 35 and again by age 45 (13). HPV testing has superior sensitivity compared to cytology and VIAC and allows for longer screening intervals after a negative test (14). Additionally, women can collect cervicovaginal specimens for testing in a process called HPV self-sampling. The use of self-collected specimens for HPV testing in screening cervical cancer among women is in line with WHO recommendations for the use of self-care interventions to promote a people-centered approach to health and well-being including for sexual and reproductive health and non-communicable diseases to achieve universal health coverage (15).

HPV testing has been used on clinician and self-collected specimens with comparable clinical accuracy (16). HPV self-sampling can potentially overcome some of the barriers that prevent women from accessing screening services (17). Evidence points to the acceptability of HPV self-sampling due to its ease of use, privacy and convenience (17). Studies conducted in limited resource settings such as Cameroon (18), Ethiopia (19), Tanzania (20) and Malawi (21) have demonstrated the acceptability of HPV self-sampling for cervical cancer screening. There is still limited HPV testing for cervical cancer screening in Zimbabwe, with the majority of work undertaken so far being led by developmental partners such as the Clinton Health Access Initiative. According to a WHO 2023 report, only 60 sites provide HPV testing services in Zimbabwe (8). The government of Zimbabwe is integrating HPV testing, including the use of HPV self-sampling, to increase screening coverage by reaching under-screened women. It is highly probable that the country will enhance screening coverage by incorporating HPV testing alongside other existing screening methods. However, since HPV testing is still a relatively new screening tool in the country, there is a shortage of evidence regarding effective delivery strategies to implement an HPV self-sampling screening programme. In order to ensure that HPV self-sampling is widely accepted and adopted by the end-users, it is crucial to develop effective delivery strategies. It is recommended that stakeholders from relevant disciplines in cervical cancer prevention and control participate in the development of these strategies.

The main aim of this study was to come up with acceptable HPV self-sampling delivery strategies using the NGT for a cervical cancer screening programme. This would aid in increasing the uptake of cervical cancer screening in rural Zimbabwe. In the past, researchers have successfully used the NGT to find the most effective delivery methods for implementing HIV self-testing programmes (22), co-creating health education programmes (23), and determining acceptable hypertension intervention packages to promote hypertension adherence (24). The findings of this study are expected to be useful to policymakers within the Ministry of Health and Child Care in Zimbabwe and concerned development partners for the design and implementation of HPV testing using self-collected specimens.

## Materials and methods

### Study setting

**Figure 1:**
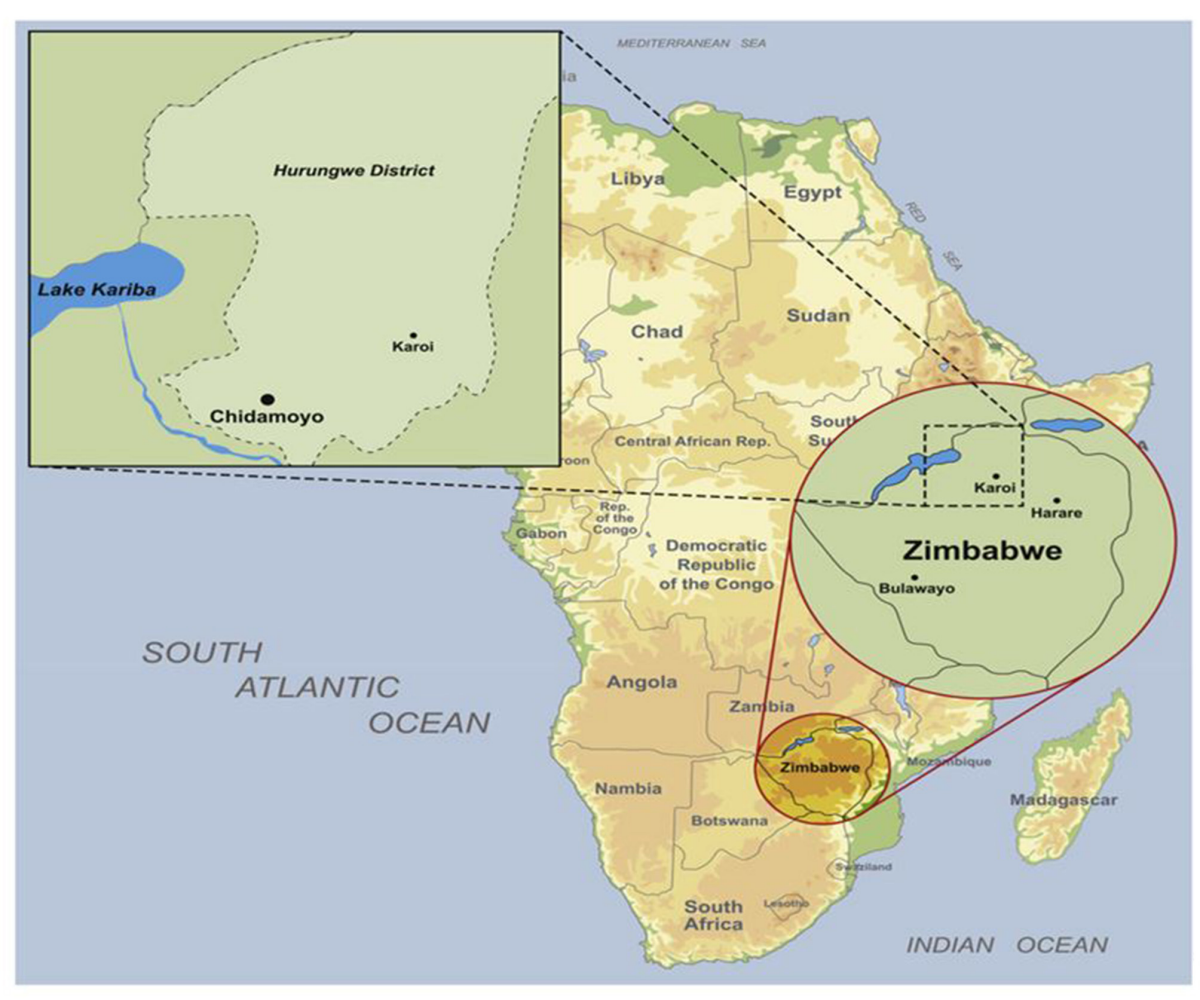
Map of Chidamoyo village in Hurungwe district (25).

The study was conducted in a village called Chidamoyo in Hurungwe rural district in Mashonaland West Province in Zimbabwe (Figure 1), with the study area defined to be Ward 13/15 which is the approximate catchment area of Chidamoyo Christian Hospital. The estimated population served by Chidamoyo Mission Hospital is 32,000 people, with ~3200 eligible women i.e., those 18 years and older (22). The researcher chose Chidamoyo village in Hurungwe as it is a rural area and traditionally rural areas have been associated with low screening coverage, poor access to or unavailability of health services (23). Additionally, it was convenient for the researcher because of a previous working relationship with the hospital administration.

### Study design

We invited key stakeholders involved in cervical cancer control and prevention in Zimbabwe. This study was part of a multiphase sequential exploratory mixed methods study to develop acceptable HPV self-sampling approaches for cervical cancer screening in Zimbabwe. The mixed methods study is underpinned by the socio-ecological model which emphasises that the interplay between individual, interpersonal, community, and societal factors influence behaviour and health outcomes (24). The results of the scoping review we conducted revealed the acceptability of HPV self-sampling and highlighted the need for more qualitative work involving stakeholders and further research on the impact of self-sampling devices (17). The systematic review highlighted the interplay of intrapersonal, interpersonal, community and health system level factors on women, health workers and policymaker's experiences and perspectives on HPV self-sampling in SSA. We combined these findings and sought to co-create acceptable delivery strategies for HPV self-sampling using the NGT.

The NGT is a highly structured face-to-face group interaction that allows participants to contribute equally and have their opinions heard by other group members. The NGT ensures that there is no domination of ideas by a single individual (25, 26). The NGT process consists of four main phases (i) silent generation- where participants generate ideas independently and write them down on a sheet of paper or sticky notes. (ii) Round robin sharing-participants take turns to share their responses without discussion or critique and these are listed on a flipchart visible to all. This process continues until all participants have shared their responses. (iii) Discussion phase-where group members discuss and ask questions in order to clarify items on the list and elaborate on their responses. During this phase, items with similar meanings are combined and duplicate items can be removed; (iv) Voting phase, here each participant is asked to prioritise the listed items by assigning ranks to them. The ranking results are then collated to produce a single list of priorities for the wider group (25).

### Study participants

The researcher invited 8 key stakeholders involved in cervical cancer screening programmes to collaborate in a co-creation workshop. The participants included four women from the target population who resided in Hurungwe, two registered nurses (one male and one female) involved in cervical cancer screening and care working at Chidamoyo Hospital, and two policymakers (one Gynaecologist in the Ministry of Health and an Epidemiologist from a development partner) and the principal investigator (as facilitator and convener) and one research assistant. Detailed characteristics of the NGT participants are presented in the next section.

### Target women

A community health worker who works closely with the women in the community used purposive sampling to identify women and recommended them to the researcher. Interest in the study was discussed between the researcher and the prospective participants taking into consideration their age and other demographic information. Four women were considered for the nominal group workshop and were informed of the workshop date and venue.

### Other key stakeholders

The researcher used a purposeful sampling strategy to invite key stakeholders to collaborate in the co-creation workshop. In our study, the term “key stakeholder” was used to refer to subject matter experts (SMEs). We defined SMEs as individuals who have expert knowledge of barriers that prevent women from accessing cervical cancer screening services and an interest in developing acceptable HPV self-sampling delivery strategies. The researcher invited these SMEs via email, printed letters and telephone calls, where an invited individual was unable to take part but suggested another person, snowball sampling was used to invite the suggested individual. Four key stakeholders were considered for the workshop and notified of the date and venue.

### Sampling strategy

A sample size of eight participants was chosen based on the researcher's assessment that the team possessed the necessary expertise and represented diverse perspectives relevant to the research question, Additionally due to the limitation in resources and the added challenge of bringing together all stakeholders at the same time, it was convenient to have 8 participants for the NGT workshop. Based on previous research, our decision for the number of participants for a nominal group workshop is influenced by the recommendations of other authors. According to Harvey and Holmes, a group consisting of 6 to 12 participants would be appropriate to gather the necessary information from each participant (25). Similarly, an NGT study conducted in Australia, which aimed to achieve consensus on graduate attributes for nurses pursuing postgraduate certification in neonatal intensive care, used a sample size of 8, similar to our study (27).

### Inclusion criteria

The study included people who fulfilled the following criteria:

- Women aged between 18 and 60 years from Chidamoyo Village, Zimbabwe
- Health workers involved in cervical cancer screening
- Programme managers/policymakers working for the Ministry of Health or development partners

### Exclusion criteria

The study excluded individuals based on the following criteria:

- Women who were non-Chidamoyo residents
- Individuals who did not give consent to participate in the study

### Nominal group process

**Figure 2:**
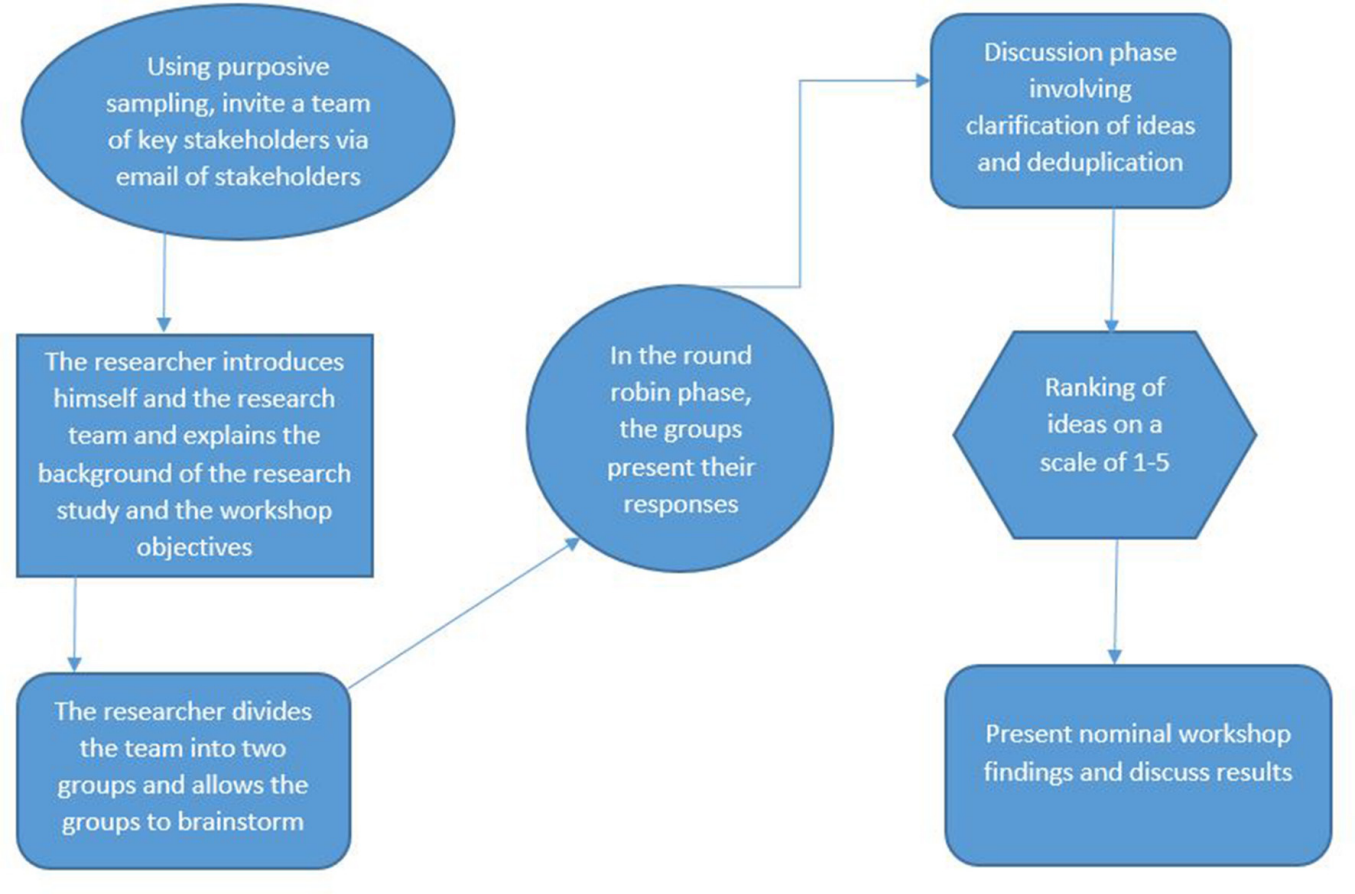
Nominal group technique (NGT) workshop workflow.

The invited stakeholders gathered on the 4^th^ of April 2023 at Chidamoyo Mission Hospital and we employed the NGT for data collection (25, 26). The workshop was conducted in two phases in a structured group discussion to achieve consensus on the priorities in response to the research questions (Figure 2): In phase 1, the stakeholders identified barriers that prevent women from accessing and utilising cervical cancer screening services. In the second phase, key stakeholders collaborated to determine acceptable HPV self-sampling delivery strategies for cervical cancer screening. The nominal group discussion was conducted in the local language. The convener of the discussion was the researcher (MD) and was assisted by a research assistant (RV). The participants were divided into two subgroups of four, with equal representation of 2 women, 1 health worker, and a policymaker in each subgroup. The questions asked to the participants at each phase were as follows:

### Phase 1

To start the workshop, the researcher (MD) posed the following question to participants: What are the barriers to access and utilisation of current cervical cancer screening services? The following steps were followed to answer this question:

### Silent brainstorming

Participants were allocated 10 minutes to write down responses on sticky notes provided with one idea on a separate note silently without discussing it with other participants. The participants were allowed to raise their hands to get the attention of the convener if they needed clarity or stationery.

### Round robin session

A total of 10 minutes was allocated to allow each group participant to present their ideas in a round-robin fashion. The ideas from the participants were grouped into similar themes and the sticky notes were put on a flipchart for presentation and discussion in the next stage of the workshop.

### Discussion and clarification of ideas

Each sub-group selected one representative to present their ideas according to the themes they had agreed upon. During this session, the audience was allowed to seek clarification and probe the presenters. The researcher with the help of the assistant collated all the ideas and highlighted similar themes. The ideas presented by each group representative were captured verbatim. The collated results were presented to the wider group as priority areas to be ranked during the ranking session.

### Ranking of ideas

The ranking process followed the approach of assigning a value to an idea according to its priority as emphasised by Delbecq et al. (28). Participants were given a short break and refreshments were provided. During this time the researcher and the research assistant printed a ranking questionnaire for each participant. Other researchers have used tools such as Google forms for the ranking stage of the NGT (29). The questionnaire was made up of barriers to access and utilisation of cervical cancer screening services as presented by the two sub-groups. The questionnaire was handed to each participant for ranking ideas using a Likert scale of 1–5 scores with 1 representing very low priority and 5 representing highest priority. The ranking process was conducted independently and without discussion. The results were collated and analysed using an Excel spreadsheet as explained in the data analysis section below.

### Phase 2

The researcher (MD) posed the following question to participants: “Which HPV self-sampling delivery strategies can help to improve women's uptake of cervical cancer screening” The steps in phase 1 were repeated in phase 2 of the workshop until the last stage of ranking the priority HPV self-sampling delivery strategies.

### Data management

During the nominal group discussions, we collected two types of data: qualitative and quantitative. We managed the two data types separately using different tools, and combined the outcomes to answer our research questions. The study assistant (RV) recorded all the qualitative data in a notebook for later analysis. For quantitative data, we entered the information into Microsoft Excel spreadsheets for further analysis. In addition to this, we received extra qualitative data from key stakeholders who provided comments on the workshop report that was sent to them immediately after the workshop.

### Data analysis

During phase 1 of the NGT, quantitative data was gathered to rank the barriers that prevent women from accessing cervical cancer screening services. Each participant provided individual scores, which were then added up to calculate a total importance score for each barrier. In phase 2, each HPV self-sampling delivery strategy was assigned a total importance score based on its effectiveness in addressing the identified barriers in phase 1. The ranking scores were on a scale of 1 to 5, with 1 being the least severe and 5 being the most severe barrier.

### Qualitative data

We conducted qualitative analysis of the top 5 ranked themes. Qualitative data from the nominal group workshop was translated into English by the researcher (MD) and the assistant (RV) who are both native Shona speakers. The transcribed text was repeatedly read to familiarise with the data. We employed the thematic analysis approach by inductively generating codes from the data presented during the discussion (30). This approach has been shown to limit researcher bias due to preconceived ideas or other theoretical perspectives (31). The first and second authors performed the data analysis.

### Ethics statement

This study was ethically reviewed and approved by two institutional review boards: University of Pretoria Faculty of Health Sciences Research Ethics Committee (approval number 548/2022) and the Medical Research Council of Zimbabwe (approval number MRCZ/A/2993). Additional written permission was sought and granted by the Ministry of Health and Childcare, Medical Directorate of Mashonaland West Province, and Chidamoyo Mission Hospital. Before participating in the study, all participants were fully informed about the study‘s background, objectives, and procedures and the researcher responded to questions regarding the study. Study participants also signed an informed consent forms to indicate their willingness to take part in the workshop. During the nominal group workshop, the participants were divided into groups with equal representation to ensure power balance and free participation. The researcher and the assistant maintained an enabling atmosphere to encourage the active participation of all stakeholders. The identities and personal information of all participants will be kept confidential and all the information shared during the discussion was anonymised to protect privacy.

## Results

All 8 invited stakeholders accepted the invitation and took part in the NGT, making it an acceptance of 100%. The stakeholders were aged between 33 and 58 years, of these, 62% were female. Fifty per cent were formally employed, three were self-employed vendors and one was unemployed. Table 1 describes the characteristics of the workshop participants.

**Table 1:** Characteristics of workshop participants.

**I. D**	**Gender**	**Marital status**	**Age**	**Highest qualification**	**Designation**	**Work experience**
P1	Female	Divorced	37	Diploma	Registered general nurse	8
P2	Female	Married	35	Ordinary level	Vendor	10
P3	Female	Married	46	Junior secondary school	Vendor	10
P4	Female	Married	52	Junior secondary school	Vendor	20
P5	Female	Married	58	Ordinary level	Unemployed	^*^
P6	Male	Single	33	Tertiary/masters	Epidemiologist	8
P7	Male	Married	40	Tertiary/masters	Obstetrician and Gynaecologist	13
P8	Male	Married	39	Diploma	Registered general nurse	5

^*^Not applicable.

### Quantitative findings

#### Phase 1

**Figure 3:**
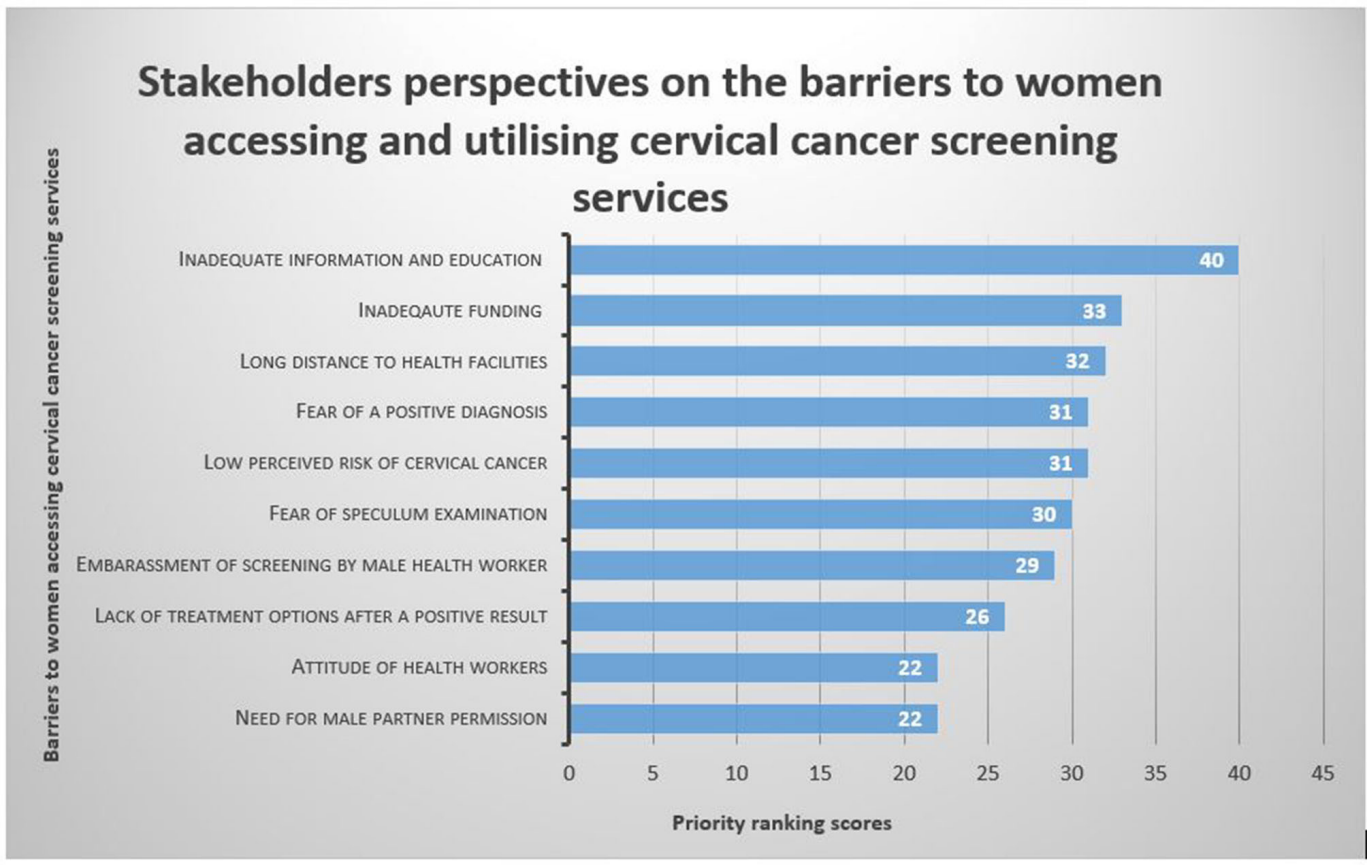
Key stakeholders' voting scores for barriers to access and utilisation of cervical cancer screening services.

Stakeholders reported 10 factors as barriers to access and utilisation of current cervical cancer screening services (Figure 3). The voting scores revealed that *inadequate information and education on cervical cancer* (with a score of 40) was the leading barrier followed by *inadequate funding for cervical cancer screening programmes* (33), *long distance to a screening health facility* (32), *fear of a positive diagnosis* (31*), low perceived risk of cervical cancer* (31), *fear of speculum examination* (30), *embarrassment of getting screened by male a health worker (30)* and *lack of treatment options after a positive result* (26). The *attitude of health workers* (22) and the *need for seeking male partner permission* were the least ranked barriers (22).

After considering the voting scores, participants identified five priority barriers to access and utilisation of available cervical cancer screening services in Chidamoyo village (Table 2). *Inadequate information and education on cervical cancer and screening methods was the highest priority barrier* (100%), followed by *inadequate funding for cervical cancer screening programmes* (82.5%), *long distance to screening health facility* (80%), *fear of a positive diagnosis* (77.5%) and the *low perceived risk of cervical cancer* (77.5%).

**Table 2:** Priority barriers total voting scores and percentages.

**Priority barriers to accessing cervical cancer screening**	**Summing by votes 1** = **low priority 5** = **highly priority**					**Total number of voting scores (number of votes × ranking score)**
	**1**	**2**	**3**	**4**	**5**	**40**
Need for male partner permission		2	6			22
Attitude of health workers	3		2	2	1	22
Lack of treatment options after a positive result	1	2	1	2	2	26
Embarrassment of screening by male health worker	1		3	1	3	29
Fear of speculum examination		1	2	3	2	30
**Low perceived risk of cervical cancer**		1	1	4	2	**31**
**Fear of a positive diagnosis**			3	3	2	**31**
**Long distance to screening health facility**		1	2	1	4	**32**
**Inadequate funding**		1	1	2	4	**33**
**Inadequate information and education**					8	**40**

The bold values represent the top rankled barriers and delivery strategies respectively.

#### Phase 2

**Figure 4:**
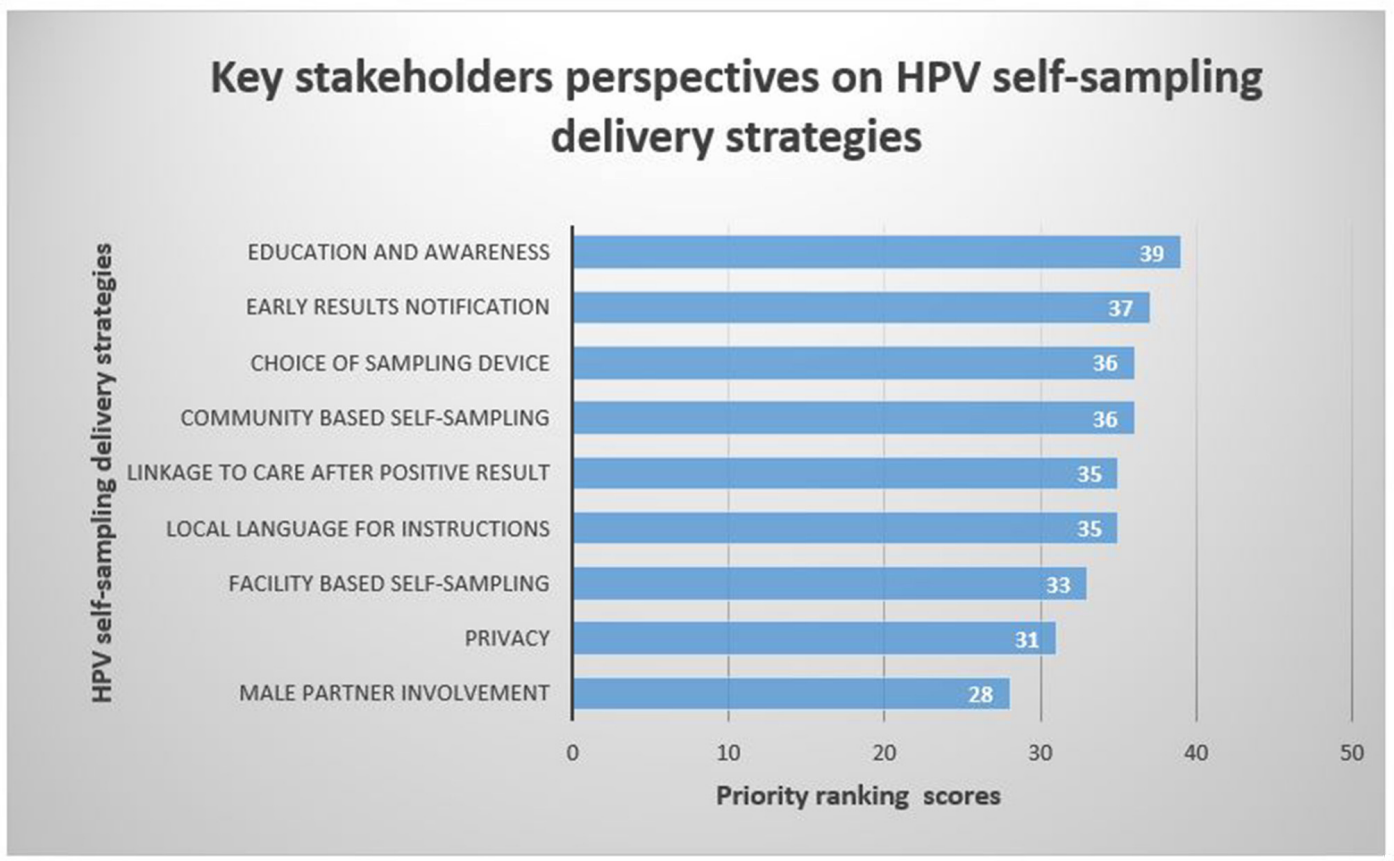
Key stakeholders' voting scores for HPV self-sampling delivery strategies for cervical cancer screening.

Stakeholders reported 9 HPV self-sampling delivery strategies for a cervical cancer screening programme (Figure 4). The voting results showed that the highest-ranked strategy for the delivery of an HPV self-sampling intervention was *education and awareness* (39). This was followed by *early results notification* (37), *community-based self-sampling* (36), *choice of sampling device* (36), *local language for instructions* (35), *linkage to care after a positive result* (35), *facility-based self-sampling* (33), *and privacy* (31) and *male partner involvement* (28) *in HPV self-sampling* were voted as the least prominent delivery strategies for the implementation of HPV self-sampling.

According to participant voting scores, the 5 HPV self-sampling delivery strategies of high priority were *Adequate education and awareness on HPV self-sampling* (97.5%), *early results notification* (92.5%), *choice of sampling device* (90%), *community-based self-sampling* (90%) and *linkage to care after positive result* (87.5%) (Table 3).

**Table 3:** Priority HPV self-sampling delivery strategies for cervical cancer screening.

**Priority HPV self-sampling delivery strategies for cervical cancer screening**	**Summing by votes 1** = **low priority 5** = **highly priority**					**Total number of voting scores (number of votes × ranking score)**
	**1**	**2**	**3**	**4**	**5**	**40**
Male partner involvement			5	2	1	28
Privacy		1	2	2	3	31
Facility based self-sampling			2	3	3	33
Local language for instructions			1	3	4	35
**Linkage to care after positive result**			1	3	4	**35**
**Community based self-sampling**			1	2	5	**36**
**Choice of sampling device**			1	2	5	**36**
**Early results notification**		1			7	**37**
**Adequate education and awareness on cervical cancer**				1	7	**39**

The bold values represent the top rankled barriers and delivery strategies respectively.

### Qualitative findings

#### Thematic analysis of the top 5 HPV self-sampling delivery strategies

The top 5 ranked priority HPV self-sampling delivery strategies as voted by the key stakeholders were (1) adequate education and awareness on HPV self-sampling (2) early results notification (3) choice of sampling device (4) community based self-sampling and (5) linkage to care. Each theme is presented below with supporting quotes.

#### Adequate education and awareness on HPV self-sampling

According to the stakeholders who participated in the workshop, the most effective delivery strategy for an HPV self-sampling screening programme was education. They suggested that the focus of education should be on providing information about cervical cancer, including its causes, prevention methods, and advantages of HPV testing using self-collected specimens over VIAC and provider-collected HPV testing. This would equip women with the necessary knowledge to make informed decisions and educate others in their communities. The women stressed that education on HPV self-sampling should be offered in their native language, such as Shona, to ensure a full understanding of the instructions and the procedure. They also requested education for their male partners to encourage their support and understanding. Community health workers were identified as key players in this initiative, as they are close to the women and wellgrounded within the communities. Education was also identified as a means to dispel misinformation and fight the stigma surrounding cervical cancer.

“Before we can do this self-sampling, may we get adequate information on how it is done so that we are able to do it correctly. It is also important for all the education and instructions on self-sampling to be conducted in (Shona) a language that we understand”

“For the majority of women, there is some satisfaction in receiving a service through a healthcare worker. Self-sampling removes this contact with the healthcare worker especially when deployed within the community. There is need to educate women that self-sampling is equally good so as to encourage uptake”

### Early results notification

Stakeholders have emphasised that early notification of results is crucial for a successful HPV self-sampling screening programme. Women experience anxiety and may discontinue the screening process if results are delayed. Compared to VIAC, results may take longer to issue to clients due to the time required to transport specimens to the laboratory. Therefore, it is essential to educate women about the advantages of HPV testing over VIAC and to make them aware that the wait is worthwhile. Stakeholders also agreed that point-of-care testing technologies with quicker turnaround times should be used. This would encourage the release of results to clients earlier. They also suggested that text-based messaging could be used to notify clients of their results. This method would be convenient since most clients have mobile phones.

**“**If we collect our own samples for cervical cancer screening, are we going to get our results early because we once collected samples for a screening programme and some of us have not received the results so I need to know if I was okay or not. Self-sampling is good but it should give us results quickly just like we get from VIAC ”

“After being screened there is anxiety about the outcome. The shorter the anxiety period the better so as to encourage uptake of services minimise loss to follow-up”

“From a practical point of view, VIAC will always have the fastest turnaround time so as we transition to HPV testing, we need to equip all the women with adequate knowledge on the benefits of HPV screening compared to VIAC so that they don't say, VIAC is better because we get results faster, Also we can also invest in point of care and near point of care technologies with quicker turnaround times compared to the big molecular platforms in central laboratories in big towns and cities”

### Choice of the sampling device

Women who participated in the workshop reported a general dislike for the metal speculum that health providers have traditionally used due to the discomfort and pain associated with its use. To make HPV self-sampling more acceptable to women, it is important to ensure that the devices used are visually appealing, easy to use, and cause little discomfort. The stakeholders emphasised that, since HPV self-sampling is a relatively new intervention, it is crucial that women have positive experiences with it, which will encourage them to share the information with their peers in the community.

**“**We hope that the thing that will be used for self-collection is not uncomfortable or painful, because I once collected my own sample and I had to stop the moment I felt some pain, now I do not know if I collected the sample properly. So, if we can have a very soft collection device which is comfortable it will be easy to perform the procedure”

“Devices causing minimal discomfort and which are visually appealing in terms of size and shape are more likely to encourage high uptake”

“I just want to know if the thing that I will use to collect is soft because the metal they put inside us is very uncomfortable, I don't like it, I am sure some of the women in here can agree with me, am I lying about the metal ladies? *No, No…they all agree with the lady* so, if a soft and painless thing is provided we are going to welcome this new method and we will tell others about it so that they also get screened”

#### Community-based self-sampling

During discussions with community-based HPV self-sampling was suggested as a crucial a delivery strategy to promote the uptake of cervical cancer screening using self-collected samples, They emphasised that accessibility was a current challenge which disproportionately affects women in rural areas, therefore, if women are afforded the opportunity to perform self-sampling screening in their communities it would be convenient for most of them. The role of community health workers in spearheading community programmes such as cervical cancer screening was highlighted and their role in educating women, raising awareness and mobilising women is key to achieving high screening coverage in communities. Stakeholders also suggested that if women would perform self-collection in the communities it was important to ensure that there would be privacy during collection. Some of the participants had this to say:

“The coverage of current screening methods is still low to achieve the coverage required to eliminate cervical cancer as a public health problem by 2030. This is because these methods cannot be scaled up to achieve that coverage due to cost, human resources, infrastructure and other challenges. Community-based self-sampling presents an opportunity for mass screening beyond the limitations of a health facility to reach underserved communities”

“This removes barriers associated with long distances to healthcare facilities and associated costs of travel, hence it is very important”

“Because some women face challenges in coming to the health facility, which may be a distance from where they stay. It would be helpful if programmes such as self-sampling can be brought to the community where the women live for convenience”

### Linkage to care after positive results

Participants were of the view that for any cervical cancer screening programme to succeed there should be follow-up on women who screen positive so that they can be triaged by another method such as VIAC. It was also highlighted that the availability of care after the screening was an important enabler for cervical cancer screening as some women reported that they were unwilling to get screened when they were unsure of getting treatment after an abnormal test.

“I don't think we will have any problems using these things to collect samples. As for me I think I want it this way instead of having that metal put inside me, however we want to know if I will be treated when found with some problems done there, because when you are HIV negative sometimes you are asked to pay for treatment but people living with HIV are treated for free. So if it stay like that I will be afraid of getting screened so I hope things change with this method of self-collection”

“HPV testing when deployed as a primary screening method requires a visual triage step for a positive result. This step will determine the treatment to be offered based on defined criteria. The goal of screening is to detect precancerous lesions and treat them. Without treatment that goal will not be met”

“When women know that something can be done for them after an abnormal result is obtained, it will encourage better uptake than if there is no plan or strategy to take care of them after a positive screen”

## Discussion

This study presents findings from a stakeholder's workshop to co-create acceptable HPV self-sampling delivery strategies for cervical cancer screening in rural Zimbabwe. Our study findings indicate that barriers at the individual, interpersonal, community and health system levels prevented women from accessing and utilising screening services. The following 5 priority barriers were identified: (1) inadequate information on cervical cancer, (2) inadequate funding, (3) long distances to health facilities, (4) fear of a positive diagnosis, and (5) low perceived risk of cervical cancer. In response, the stakeholders proposed delivery strategies to overcome some of the identified barriers, for instance, education and awareness was identified as the highest-ranked strategy to overcome the lack of knowledge, low perceived risk of cervical cancer and fear of a positive diagnosis while community-based self-sampling was proposed as a strategy to overcome long distances to health facilities. Our findings on barriers to access and utilisation of screening services are corroborated by studies that were conducted in Zimbabwe (11, 16, 23, 32) and other countries in SSA (33, 34). The lack of education and information on cervical cancer and screening methods was considered a significant barrier by the stakeholders in the current study. Women in this study reported that compared to other diseases such as HIV and TB, there were no widespread campaigns and awareness on cervical cancer and this likely contributed to the lack of knowledge on the disease. Similar findings were reported in previous studies (35, 36). A qualitative study to explore community knowledge, facilitators and barriers to cervical cancer screening in rural Uganda revealed a belief among women that screening should be accessed at the onset of symptoms (35). Additionally, a Swedish study revealed that women lacking education had positive perceptions towards screening but prioritised other things in their lives, particularly when asymptomatic (36). This underscores the need for extensive education of women on cervical cancer prevention and the importance of seeking screening services early before the onset of symptoms when the cancer would probably have advanced. According to stakeholders, women were fearful of the pain and discomfort associated with the use of a metal speculum during pelvic exams. This is further emphasised by a qualitative study conducted in rural Kenya where women expressed a preference for self-sampling over pelvic exams due to the invasive and painful nature of the latter (37). We identified the lack of funding for cervical cancer control and prevention programmes and inaccessible health facilities as health system-level barriers. Petersen and colleagues reported the significant impact of factors such as (low budgetary support), infrastructure, and health workers on the accessibility and utilisation of cervical cancer screening services by women in limited resource settings (38).

### HPV self-sampling delivery strategies

The highest-ranked strategies in our study included: education and awareness on HPV self-sampling, early results notification, community-based HPV self-sampling, choice of the sampling device, and linkage to care. Education and awareness was the highest-ranked delivery strategy which is important in overcoming barriers such as the lack of education, fear of positive diagnosis and the perceived low risk of cervical cancer. A systematic review conducted in Uganda (39) identified the fear of screening procedures as the major barrier to the uptake of screening services, but the authors revealed that this was due to misconceptions and myths which could all be dispelled by proper education of women. Therefore the role of education in overcoming many of the barriers at the individual level cannot be ignored. Delivery of education through peer educators has also been shown to increase the acceptability and uptake of cervical cancer screening (40, 41). According to findings from a systematic review by Makadzange et al. the use of peer educators and culturally sensitive and tailored material significantly impacted the delivery of educational messages for cervical cancer prevention to the target population in Africa (40). Similarly, a study conducted in India also supports using culturally appropriate educational material and interventions to reach communities and promote the uptake of cervical cancer screening especially among rural communities where the lack of education is the major hurdle to increased screening uptake among women (41).

The early delivery or notification of results was suggested as an important strategy for an HPV self-sampling screening programme. Stakeholders emphasised the need for early notification to avoid the anxiety associated with one not knowing their result, further contributions on this strategy highlighted the importance of early results notification to minimise the loss to follow-up and to encourage women participation in cervical cancer screening in the future. Not much is known on how the wait for HPV results affect women particularly in low income countries where VIA has been the main screening modality that ensures same day results. Considering the extensive mobile network coverage in Zimbabwe and that almost every person owns a mobile phone, text based messaging will be the most ideal notification method in Zimbabwe. A study in rural Tanzania that employed text messaging for results notification revealed the method to be acceptable and it encouraged women to attend a follow-up appointment after receiving HPV results (42). Additionally, leveraging on point-of-care diagnostic technologies which were widespread throughout Zimbabwe's districts during the COVID-19 pandemic may encourage faster turnaround times leading to early results notification for women in rural areas as compared to referring specimens for laboratory testing in towns and cities (43).

Stakeholders agreed that for HPV self-sampling to be an appealing screening method, women who screen positive for HPV must be easily linked to care. Current screening methods using VIAC in Zimbabwe encourage same-day treatment after an abnormal test and therefore it is critical to ensure that women get treatment services near them if they are eligible so that they do not lose trust in the HPV self-sampling screening method. A study by McRae et al. in which women were transitioning from cytology to HPV testing reported women's frustration with the lack of adequate treatment services after an HPV test because they were used to getting treatment and care without delay when undergoing cytology screening (44). There is a need for adequate education of women on the procedures relating to HPV testing such as the triaging of women who screen positive to determine their eligibility for treatment so that they appreciate the delay in getting care is necessary. It is worth noting that the success of any screening method ultimately rest on the identification of those at risk and their treatment, it is therefore crucial that such services are easily accessible to women in need.

The choice of sampling device was identified as a priority by stakeholders. Studies conducted in some countries in SSA highlighted the ease of use and comfort women experienced during self-sampling, making it easy for them to prefer future screening using self-collected devices (45, 46). Stakeholders in this study emphasised the need for visually appealing devices that cause minimal discomfort and less pain to ensure that women have a positive experience after screening. This has been shown to encourage the willingness to participate in future screening and to spread positive messages to peers and family members which in turn increases screening coverage (47). Megersa et al. (48) revealed that the choice of sampling device was a very important aspect of an HPV self-sampling intervention as the fear of the Evalyn brush in their study affected the quality of the sample collected and participants were less willing to use the brush next time for self-sampling. In the Zimbabwean context where resources are limited, there is likely going to be a single type of device for self-sampling. It is, therefore, vital to decide on the most preferred device before implementing the intervention. Adequate education in the local language including the use of pictures and videos can improve understanding of the self-sampling process ultimately increasing self-efficacy in performing self-sampling. Stakeholders in our study strongly recommended community-based HPV self-sampling approaches. Studies conducted in Zimbabwe (22) and Malawi (21) have all demonstrated the utility of bringing HPV testing closer to people. Likewise, in Cameroon, campaigns for HPV self-sampling in the community have proven feasible and cost-effective in increasing screening coverage, as demonstrated by same-day screening and treatment initiatives (18). In Zimbabwe, we recommend that the Ministry of Health and Child Care and development partners take advantage of traditional gatherings for women, such as “China cheMadzimai”, a gathering of religious women on Thursdays to reach women and promote HPV self-sampling cervical cancer screening.

The collaboration of different stakeholders enabled different delivery strategies for implementing HPV self-sampling to be heard. The proposed approaches have the potential to promote demand and increase the acceptability of HPV self-sampling in Zimbabwe and other similarly resource-limited areas. To determine the most preferred delivery strategies for HPO self-sampling among women, we suggest employing the discrete choice experiment with a large sample size from the same study setting.

### Strengths and limitations of the study

The collaboration of different stakeholders to co-create delivery strategies for an HPV self-sampling screening intervention is a notable strength of this study. Another strength of this study was the involvement of rural women as stakeholders. Rural women are disproportionately affected by cervical cancer due to their low socioeconomic status and lack of access to healthcare facilities and capturing their perspectives is vital in tailoring interventions to improve cervical cancer screening uptake not only in Zimbabwe but also in similarly low-resource rural settings globally. The collection of both quantitative and qualitative data allowed the ranking of strategies and allowed the researchers to obtain qualitative data. The themes were not selected a priori but rather actively constructed by the group. Future researchers may replicate the methods for co-creation purposes. A limitation of our study was the absence of other important stakeholders such as community and traditional leaders, community health workers and laboratory personnel during the workshop to offer their perspectives on barriers and potential delivery strategies. It would have been beneficial to involve these individuals to ensure that no crucial insights were overlooked. Although our sample size was small, we managed to capture diverse perspectives from the present stakeholders. However, it is possible that the women felt intimidated and were unable to participate freely due to the presence of familiar faces from their local hospital who were also health workers, even though the researcher ensured that participation was voluntary.

## Conclusion

The purpose of the study was to develop effective HPV self-sampling delivery strategies for cervical cancer screening in rural Zimbabwe through co-creation with different stakeholders. Our research has demonstrated the effectiveness of the NGT for reaching a consensus on the barriers to access and utilisation of available screening services and identifying potential delivery strategies for HPV self-sampling to overcome identified barriers. The stakeholders identified and ranked them according to their priority in the following order: (1) education and awareness, (2) early results notification (3) choice of sampling device (4) community-based self-sampling and (5) and linkage to care. We anticipate that these proposed delivery strategies will be used by the Ministry of Health and Child Care, development partners and other relevant stakeholders to design an effective HPV testing screening programme using self-collected specimens in rural Zimbabwe.

### Recommendations

After the success of NGT in identifying delivery strategies for HPV self-sampling and ranking them according to their priority, we recommend more stakeholder involvement in designing and implementing a national programme for HPV testing using self-collected specimens. To achieve this, we suggest involving the government, community health workers, traditional and community leaders, youth advocates, laboratory personnel, and supply chain experts. To ensure better access and utilisation of cervical cancer screening services, we also recommend an education programme targeting rural women, male partners, and community leaders on HPV-based cervical cancer screening using self-collected specimens. Lack of education was identified as the main barrier to accessing these services. The education programme can be championed by community health workers who work closely with women and the wider community on a day-to-day basis. To improve HPV testing turnaround times, we suggest leveraging point-of-care technologies used for COVID-19 testing and using text messaging for result notifications. Further research is needed in Zimbabwe to evaluate the impact of waiting for results on women's willingness to participate in cervical cancer screening with HPV testing. Also, before implementing HPV self-sampling screening, there is need to conduct a follow-up study to determine rural women's preferences for delivery strategies using a larger sample size with a discrete choice experiment survey.

## Data availability statement

The raw data supporting the conclusions of this article will be made available by the authors, without undue reservation.

## Ethics statement

The studies involving humans were approved by University of Pretoria Faculty of Health Sciences Research Ethics Committee and the Medical Research Council of Zimbabwe. The studies were conducted in accordance with the local legislation and institutional requirements. The participants provided their written informed consent to participate in this study. Written informed consent was obtained from the individual(s) for the publication of any potentially identifiable images or data included in this article.

## Author contributions

MD: Conceptualization, Data curation, Investigation, Methodology, Project administration, Resources, Visualization, Writing—original draft. TD: Conceptualization, Investigation, Supervision, Writing—review & editing, Methodology. GM: Writing—review & editing. TC: Writing—review & editing. RM: Writing—review & editing. MF: Writing—review & editing. TM-T: Conceptualization, Investigation, Methodology, Supervision, Writing—review & editing.
